# Optimization of CRISPR/Cas9‐mediated gene disruption in *Xenopus laevis* using a phenotypic image analysis technique

**DOI:** 10.1111/dgd.12778

**Published:** 2022-04-12

**Authors:** Mikio Tanouchi, Takeshi Igawa, Nanoka Suzuki, Makoto Suzuki, Nusrat Hossain, Haruki Ochi, Hajime Ogino

**Affiliations:** ^1^ Amphibian Research Center/Graduate School of Integrated Sciences for Life Hiroshima University Hiroshima Japan; ^2^ Institute for Promotion of Medical Science Research, Faculty of Medicine Yamagata University Yamagata Japan

**Keywords:** CRISPR/Cas9, genome editing, *Xenopus*

## Abstract

The CRISPR/Cas9 method has become popular for gene disruption experiments in *Xenopus laevis*. However, the experimental conditions that influence the efficiency of CRISPR/Cas9 remain unclear. To that end, we developed an image analysis technique for the semi‐quantitative evaluation of the pigment phenotype resulting from the disruption of *tyrosinase* genes in *X. laevis* using a CRISPR/Cas9 approach, and then examined the effects of varying five experimental parameters (timing of the CRISPR reagent injection into developing embryos; amount of Cas9 mRNA in the injection reagent; total injection volume per embryo; number of injection sites per embryo; and the culture temperature of the injected embryos) on the gene disruption efficiency. The results of this systematic analysis suggest that the highest possible efficiency of target gene disruption can be achieved by injecting a total of 20 nL of the CRISPR reagent containing 1500 pg of Cas9 mRNA or 4 ng of Cas9 protein into two separate locations (10 nL each) of one‐cell stage embryos cultured at 22°C. This study also highlights the importance of balancing the experimental parameters for increasing gene disruption efficiency and provides valuable insights into the optimal conditions for applying the CRISPR/Cas9 system to new experimental organisms.

## Graphical abstract

Using a novel phenotypic image analysis technique, we optimized experimental conditions for CRISPR/Cas9‐mediated gene disruption in *Xenopus laevis*. This study highlights the critical balance on the experimental parameters for increasing gene disruption efficiency, and provides valuable insights into setting conditions for applying the CRISPR/Cas9 system to new experimental organisms.

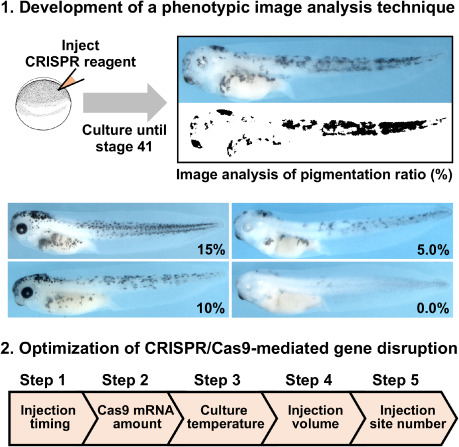

## 1 INTRODUCTION

Clustered regulatory interspaced short palindromic repeats/CRISPR associated protein 9 (CRISPR/Cas9) has become a popular genome editing tool in *Xenopus laevis* (Aslan et al., 2017; Banach et al., 2017; Bharathan & Dickinson, 2019; Carr et al., 2021; Delay et al., 2018; Delay et al., 2019; Ezawa et al., 2021; Feehan et al., 2017; Jaffe et al., 2016; Kato et al., 2021; Kim et al., 2021; Ledford et al., 2017; Martin et al., 2020; Naert et al., 2020; Ochi et al., 2017; Pickett et al., 2017; Smith et al., 2020; Square et al., 2020; Tandon et al., 2017; Tsujioka et al., 2017; Viet et al., 2020; Wang et al., 2015; Wen et al., 2019; Wyatt et al., 2021). Importantly, most studies to date delivered the CRISPR reagents using a microinjection into fertilized eggs. As the cleavage of the injected egg proceeds, target gene disruption occurs in each cell lineage, such that the relative amount of Cas9/sgRNA complex decreases as the amount of nuclear genomic DNA increases due to replication (Smith et al., 2020). Hence it is difficult with this technique to disrupt target genes uniformly and efficiently throughout the founder embryo, despite its strong need in this species whose generational time (1–2 years) is too long to obtain the homozygous offspring quickly. Furthermore, the experimental conditions that may influence the disruption efficiency, such as the timing of the CRISPR reagent injection after the fertilization, or the amount of Cas9 mRNA injected, vary greatly among previous studies, and the optimum conditions for achieving highly efficient gene disruption remain unclear.

**FIGURE 1:**
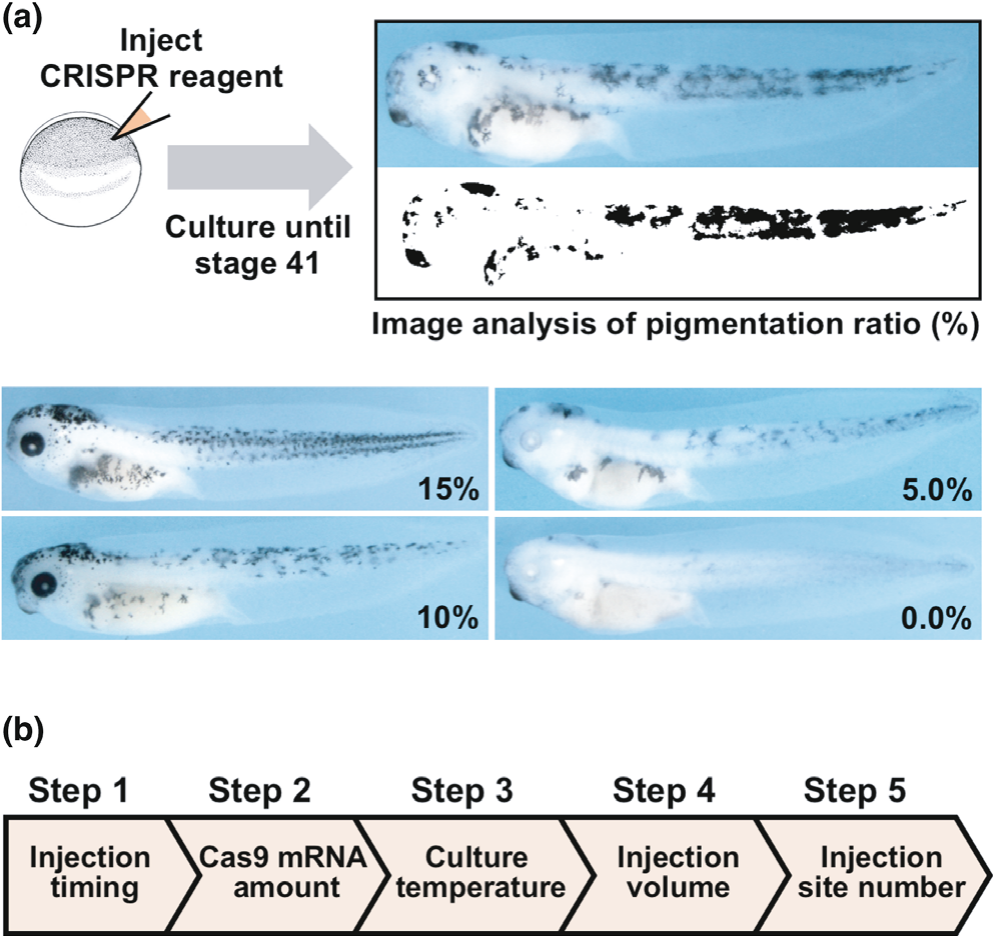
Experimental design for identifying conditions that increase gene disruption efficiency. (a) Semi‐quantitative image analysis of the pigment phenotype (upper panels) and representative embryos with specific percentages of pigmented regions (lower panels). (b) Flow chart of the step‐by‐step analyses of the five experimental parameters expected to influence gene disruption efficiency

Here we systematically examined the effects of varying multiple experimental conditions to achieve the highest gene disruption efficiency by injecting CRISPR reagents into fertilized *X. laevis* eggs. As the target genes, we chose two *tyrosinase* homoeologs, *tyr.L* and *tyr.S*, whose simultaneous disruption results in melanin loss from the pigment cells (Wang et al., 2015). This previous study only evaluated the degree of melanin loss qualitatively by visual inspection. Therefore, we developed a semi‐quantitative image analysis technique to evaluate the resulting phenotypes with higher accuracy (Figure 1a). Using this technique, we examined the combinatorial effects of varying five experimental parameters on the gene disruption efficiency in a step‐by‐step manner (Figure 1b). In Step 1, we examined the effects of varying the injection timing. This was inspired by the finding that the I‐SceI meganuclease method can improve the transgenesis efficiency by introducing transgenesis reagents as early as possible after fertilization (Ogino et al., 2006). In step 2, we examined the effects of varying the amount of Cas9 mRNA per embryo, as it has been shown that increasing the Cas9 mRNA amount to a certain level improves gene disruption efficiency in *X. tropicalis* (Blitz et al., 2013; Nakayama et al., 2013). Although previous studies have cultured injected embryos at various temperatures ranging from 12 to 22°C, there are no reports examining the effects of varying the culture temperature while keeping all other experimental conditions constant (Banach et al., 2017; Carr et al., 2021; Feehan et al., 2017; Kato et al., 2021; Smith et al., 2020; Tsujioka et al., 2017: Viet et al., 2020). Therefore, in step 3, we examined the effects of varying the embryo culture temperature post injection. Finally, in steps 4 and 5 we examined the effects of varying the total volume of injected reagent per embryo and the number of injection sites per embryo. These differences may influence gene disruption efficiency by changing the degree of diffusion of the injected reagents in the embryo.

## 2 MATERIALS AND METHODS

### 2.1 Preparation of Cas9 mRNA and sgRNAs

Capped mRNA encoding the human codon‐optimized *S. pyogenes* Cas9 nuclease was transcribed from pCS2 + hSpCas9 (Ansai & Kinoshita, 2014). *tyr.L*‐sgRNA and *tyr.S*‐sgRNA were transcribed from the template plasmids generated by introducing annealed oligonucleotide pairs (*tyr.L*, 5′‐TAGGGTCGATGATAGAGGAC‐3′ and 5′‐ AAACGTCCTCTCTATCATCGAC‐3′; *tyr.S*, 5′‐TAGGCCCGTAGCAGAGCTGGTG‐3′ and 5′‐AAACCACCAGCTCTGCTACGGG‐3′) into pDR274, respectively (Hwang et al., 2013). The target sequences of *tyr.L*‐sgRNA and *tyr.S*‐sgRNA were 5′‐ GTCGATGATAGAGAGGAC‐3′ and 5′‐ CACCAGCTCTGCTACGGG‐3′, as described earlier (Wang et al., 2015).

### 2.2 Microinjection into *X. laevis* embryos

Artificial fertilization was carried out as previously described (Sive et al., 2000). The resulting eggs were only used when the pigment contraction was confirmed in more than 95% of eggs after mixing with the sperm slurry. The eggs were then dejellied, transferred into the injection medium whose temperature was pre‐adjusted for each experimental condition, and injected along with CRISPR reagents into the marginal zone of the animal hemisphere. The one‐cell stage injection was completed 30 min after the fertilization at all the temperature conditions. The injected embryos and their uninjected sibling embryos were used for subsequent analysis only when both groups reached gastrula stages without any apparent differences in the rate of normally gastrulating embryos. The amounts of injected eGFP mRNA (lineage tracer), *tyr.L*‐sgRNA, and *tyr.S*‐sgRNA were fixed to 50, 300, and 300 pg per embryo, respectively.

### 2.3 Analyses of pigment phenotypes and *tyr* disruption efficiencies

**FIGURE 2:**
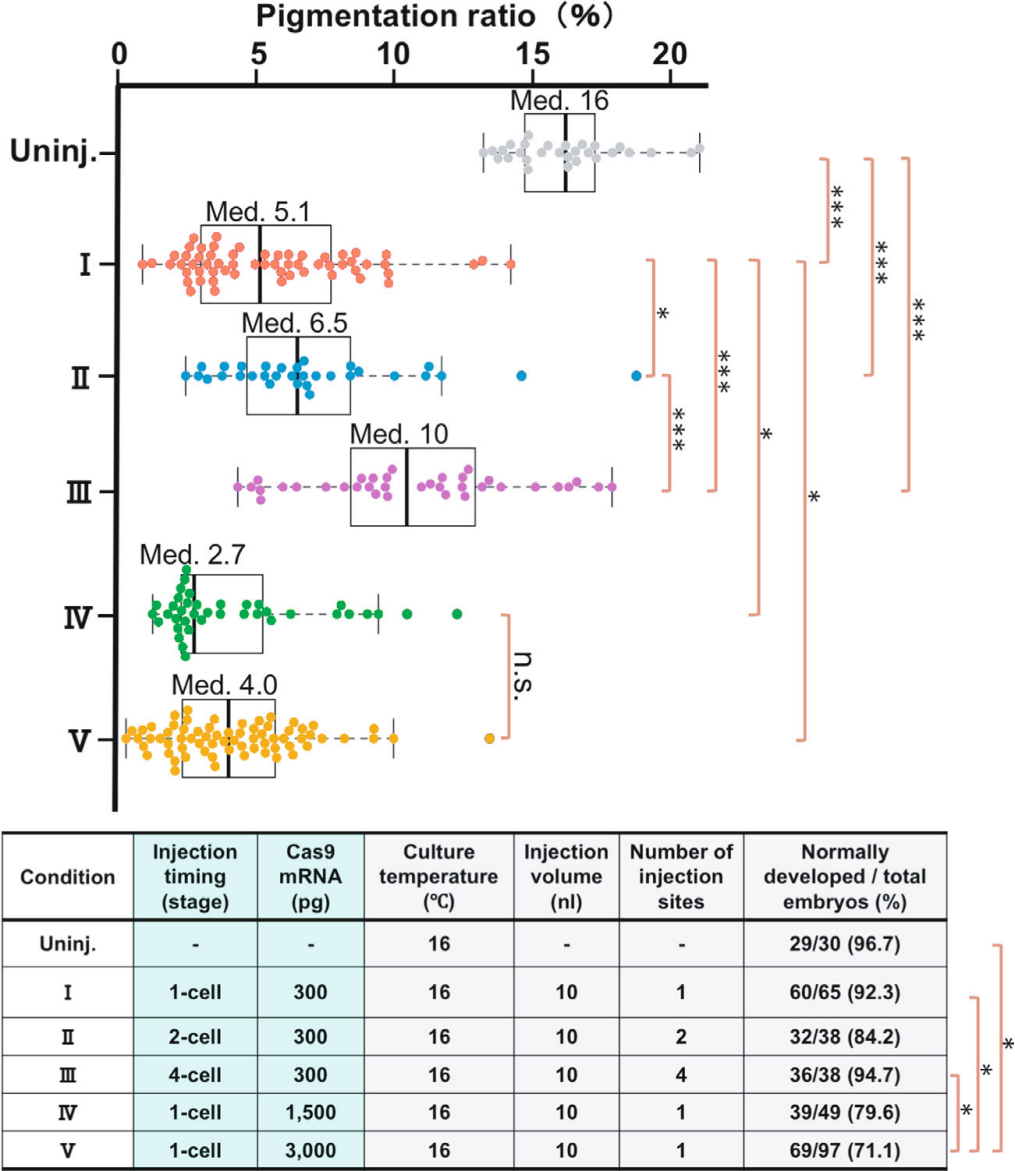
Effects of varying the injection timing and the amount of injected Cas9 mRNA per embryo. A box‐and‐whisker plot showing pigmentation ratios of the uninjected embryos (Uninj.) and the embryos generated under the experimental conditions shown in the Table (I, II, III, IV, and V) as dots. In each sample group, vertical lines indicate the minimum, first quartile, median (Med.), third quartile, and maximum values excluding outliers beyond the whisker end, respectively, from left to right. The upper limit of the whisker is 1.5 times the box length. Only significant differences in the rates of normally developed embryos are shown in this and other tables. ***p < .001; *p < .05; and “n.s.” (not significant)

**FIGURE 3:**
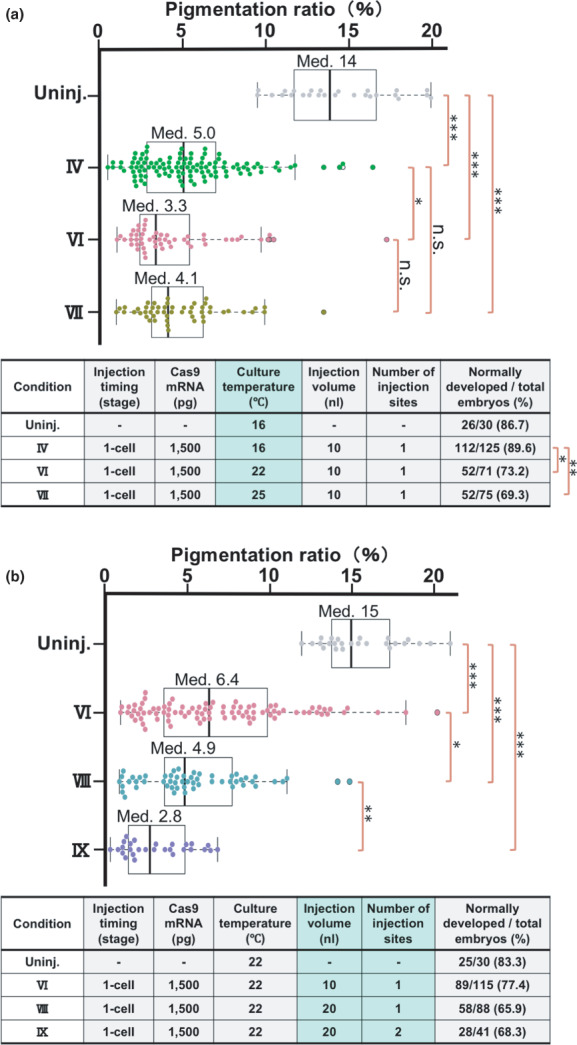
Effects of varying the culture temperature of the injected embryos, the volume of the injected reagents, and the number of sites injected. (a) A box‐and‐whisker plot showing the pigmentation ratio of uninjected embryos cultured at 16°C and the embryos generated under the experimental conditions shown in the Table (IV, VI, and VII). (b) A box‐and‐whisker plot showing the pigmentation ratios of the uninjected embryos and the embryos generated under the experimental conditions shown in the Table (VI, VIII, and IX). **p < .01

**FIGURE 4:**
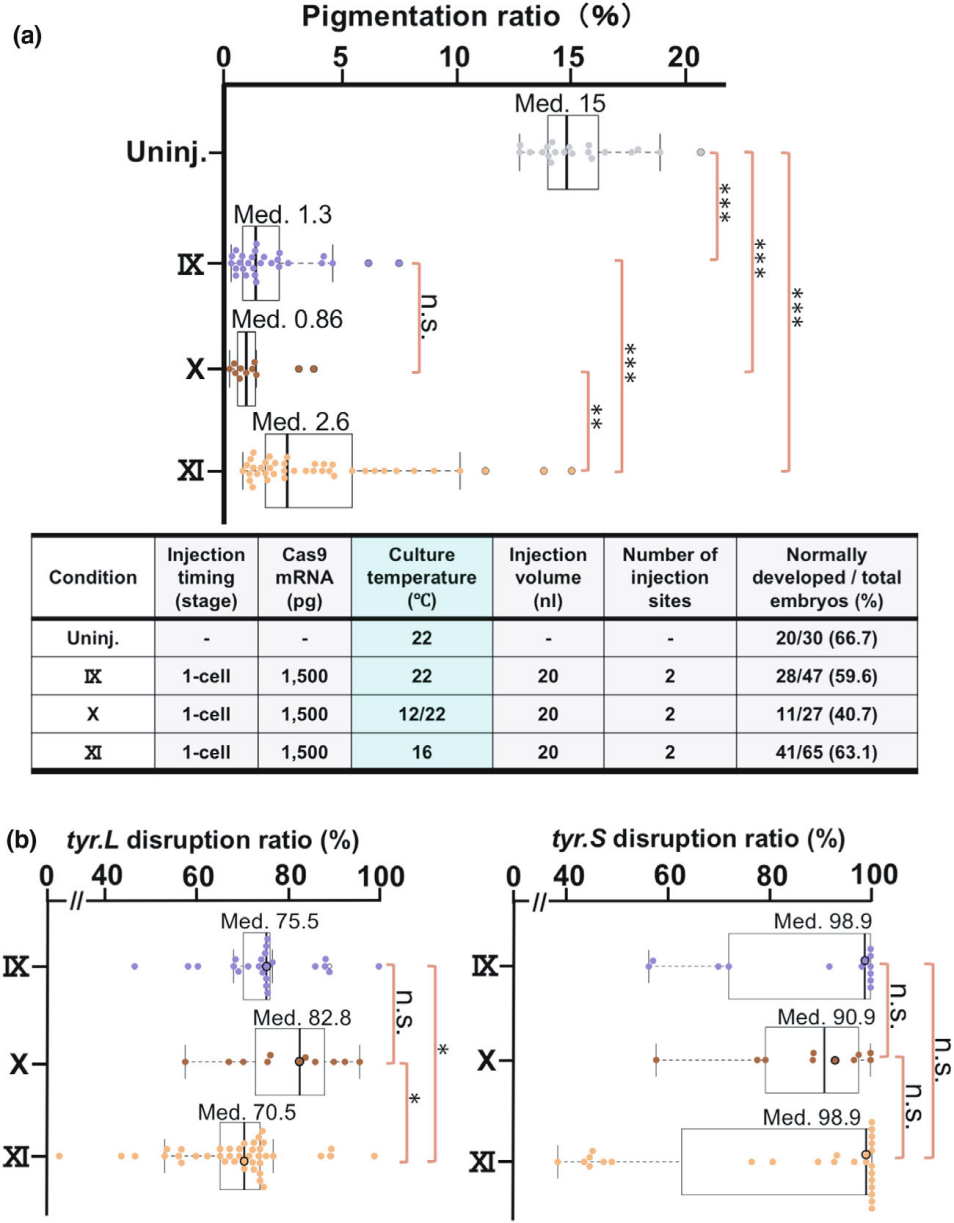
Reassessment of culture conditions for injected embryos. (a) A box‐and‐whisker plot showing the pigmentation ratios of the uninjected embryos cultured at 22°C and the embryos generated under the experimental conditions shown in the Table (IX, X, and XI). (b) The left and right box‐and‐whisker plots show the disruption ratios of *tyr.L* and *tyr.S* in embryos generated under the conditions IX, X, and XI, as dots, respectively. Since the plots were generated only with the disruption ratios whose certainty (R‐squared) were greater than the recommended value (0.8) in the ICE analysis, the plotted dot numbers for *tyr.L* and *tyr.S* are different. The raw output data of the ICE analysis corresponding to black‐lined dots are shown in Figure S4

We performed a phenotypic image analysis by calculating the percentage of pigmented area to total body area on both sides of stage 41 embryos (pigmentation ratio) using Image J (Figure 1a). Statistical analyses of the pigment‐phenotype or genotype differences in Figures 2, 3, 4 and S3 were performed using the Wilcoxon rank‐sum test with the Holm p‐adjustment included in R version 3.6.3 (http://www.R-project.org).

The *tyr* disruption efficiency was analyzed by direct sequencing of the target regions amplified from the genomic DNA extracted from each manipulated embryo, using the Inference of CRISPR Edits (ICE) tool (https://www.synthego.com). The amplification reaction was performed using primer pairs for *tyr.L* (5′‐AGATTCCCAGTCCCTTAGATCAGG‐3′ and 5′‐ATTGGAGCCGTTATTCATCTGAGC‐3′) and *tyr.S* (5′‐ TGATTCCCAGTCCCTTAGAGCAGA‐3′ and 5′‐ CTGCAAACATAGGGTTGGAGCCA‐3′).

We compared the experimental results only within embryos obtained by successive fertilization on the same day using the same female and male pair to minimize data fluctuation. To examine the frequency of developmental abnormalities unspecific to the *tyr* disruption, we scored the number of embryos with normal external morphology, excluding the pigment phenotype, and the total number of analyzed embryos in each sample group, along with a percentage of the embryos with normal external morphology (shown as “normally developed/total embryos (%)” in tables). We then analyzed the obtained rates of normally developed embryos using the Fisher's exact test in Figures 2, 3, 4.

## 3 RESULTS AND DISCUSSION

In Step 1, we injected the mixture of Cas9 mRNA, *tyr.L* sgRNA, and *tyr.S* sgRNA into one‐cell, two‐cell, or four‐cell stage embryos (Figure 2, table). The amount of Cas9 mRNA injected into each embryo was adjusted to 300 pg as reported previously (Wang et al., 2015). For the one‐cell stage embryos, we introduced 10 nL of the RNA mixture in a single injection (Condition I). For the two‐cell and four‐cell stage embryos, we injected 5 and 2.5 nL of the RNA mixture into each blastomere, respectively, to adjust the total volume of the injected RNA mixture per embryo to 10 nL, as previously described (Conditions II and III, respectively) (Banach et al., 2017; Ezawa et al., 2021). After the injection, the embryos were cultured at 16°C before being subjected to the phenotypic image analysis. The median pigmentation ratios of the resulting Condition I, II, and III embryos were 5.1%, 6.5%, and 10%, respectively, whereas the median of the uninjected embryos was 16% (Figure 2, box‐and‐whisker plot). The pigmentation ratio was significantly lower in the Condition III embryos compared to the uninjected embryos, in the Condition II embryos compared to the Condition III embryos, and in the Condition I embryos compared to the Condition II embryos. These results indicate that the one‐cell stage injection is preferable for maximizing the gene disruption efficiency, which is consistent with the previous study (Smith et al., 2020).

In Step 2, increasing amounts of Cas9 mRNA were injected (1500 or 3000 pg) together with the unchanged amounts of sgRNAs into the one‐cell stage siblings of the embryos used for the Step 1 experiments (Conditions IV and V, respectively; Figure 2, table). The median pigmentation ratios of the Condition IV and V embryos were 2.7% and 4.0%, respectively. The pigmentation ratio was significantly lower in the Condition IV and V embryos compared to the Condition I embryos injected with 300 pg of Cas9 mRNA (Figure 2, box‐and‐whisker plot). However, there were no significant differences between the Condition IV and V embryos. The rate of normally developed embryos (evaluated by other than pigment phenotype) in the Condition V embryos (69/97) was significantly lower than that in the uninjected embryos (29/30), whereas no significant differences were detected between those of the Condition IV embryos (39/49) and the uninjected embryos (Figure 2, table). These results indicate that the gene disruption efficiency increases as the Cas9 mRNA amount increases but reaches a plateau when the amount exceeds around 1500 pg, with any further increase in Cas9 mRNA affecting embryonic development. Based on these findings, we fixed the injection timing to the one‐cell stage and the amount of Cas9 mRNA to 1500 pg per embryo in the subsequent experiments. Importantly, although we used hSpCas9 in this study, the optimal amount may need to be re‐adjusted if one uses the Cas9 with a different codon usage (Tandon et al., 2017).

In Step 3, we performed the injections under the condition fixed by Step 2 and cultured the injected embryos at three different temperatures, 16, 22, and 25°C (Condition IV, VI, and VII, respectively; Figure 3a, table). The embryos in Steps 1 and 2 were cultured at 16°C, whereas 25°C is close to the highest culture temperature that *X. laevis* embryos can tolerate (Khokha et al., 2002). We found that varying the temperature did not influence the pigmentation ratios of the uninjected embryos (Figure S1). The median pigmentation ratios of the Condition IV, VI, and VII embryos were 5.0%, 3.3%, and 4.1%, respectively (Figure 3a, box‐and‐whisker plot). The pigmentation ratio was significantly lower in the Condition VI embryos compared to the Condition IV embryos. This finding may reflect the temperature dependency of Cas9 nuclease activity, which increases as the temperature increases and reaches peak activity around 39°C (Xiang et al., 2017). However, there was no significant difference between the Condition IV and VII embryos, implying that the higher culture temperature enhances the Cas9 activity but shortens the essential time window at the early stages by accelerating embryonic development. The rate of normally developed embryos in the Condition VI embryos (52/71) and that in the Condition VII embryos (52/75) were significantly lower than that in the Condition IV embryos (112/125) (Figure 3a, table). These results indicate that the 22°C culture slightly decreases the rate of normally developed embryos but increases the gene disruption efficiency, whereas a further temperature increase does not improve the efficiency. Thus, we fixed the culture temperature at 22°C for the subsequent experiments.

In Step 4, we performed experiments under the condition fixed by Step 3, but varied the total volume of the injected reagent per embryo. The first group of embryos was injected with 10 nL of the Cas9 mRNA/*tyr* sgRNAs mixture like the experiments performed in Step 3 (Condition VI; Figure 3b, table). For the second group, we diluted the RNA mixture by adding an equal volume of purified water and injected 20 nL of the resulting reagent per embryo (Condition VIII; Figure 3b, table). The median pigmentation ratios of the Condition VI and VIII embryos were 6.4% and 4.9%, respectively (Figure 3b, box‐and‐whisker plot). The pigmentation ratio was significantly lower in the Condition VIII embryos compared to the Condition VI embryos.

In Step 5, we examined the effects of varying the number of injection sites using the siblings of the embryos used for the Step 4 experiments. We kept the total volume of the injected RNA mixture to 20 nL as in the Condition VIII, but injected it separately into two diagonal locations of the animal hemisphere, 10 nL each (Condition IX; Figure 3b, table). The median pigmentation ratio of the Condition IX embryos was 2.8%. Their pigmentation ratio was significantly lower than that of the Condition VIII embryos (Figure 3b, box‐and‐whisker plot). Regarding the rates of normally developed embryos, no significant difference was detected among the Condition VI, VIII, and IX embryos. The results of the Steps 4 and 5 experiments indicate that increasing the total volume of the RNA mixture to 20 nL and dividing it into two injections of 10 nL each can further improve gene disruption efficiency, without a significant decrease in the rate of normally developed embryos.

We previously found that the I‐SceI meganuclease method increases the transgenesis efficiency when the manipulated embryos are incubated at 12–13°C until the two‐ to four‐cell stage (Ogino et al., 2006). To examine whether such low‐temperature incubation can further improve gene disruption efficiency, we injected the Cas9 mRNA/*tyr* sgRNAs mixture under the experimental conditions optimized by Step 5 and cultured the resulting embryos under three different temperature conditions (Figure 4a, table). The first group of embryos was cultured at a constant temperature of 22°C after the injection, as in the Step 5 experiments (Condition IX). The second group was initially cultured at 12°C for 6 hours (until the two‐ to four‐cell stage) and subsequently at 22°C (Condition X). The third group was cultured at a constant temperature of 16°C after the injection (Condition XI). The median pigmentation ratios of the Condition IX, X, and XI embryos were 1.3%, 0.86%, and 2.6%, respectively (Figure 4a, box‐and‐whisker plot). The pigmentation ratio was significantly lower in the Condition IX and X embryos compared to the Condition XI embryos. There were no significant differences in the pigmentation ratios between the Condition IX and X embryos. Furthermore, no significant differences were detected in the rates of normally developed embryos among the Condition IX, X, and XI embryos.

The significant differences in pigmentation ratios among the sample groups were reproduced by comparing the mean values obtained from the experiment shown in Figure 4a with two additional independent experiments using embryos from different female and male pairs (Figure S2). Furthermore, pigmentation ratios comparable to that of the Condition IX embryos were obtained when the Cas9 mRNA was replaced with increasing amounts of Cas9 protein (Integrated DNA Technologies) in the Condition IX, indicating the efficacy of the conditions optimized here (Figure S3).

Next, we examined the actual disruption ratios of *tyr.L* and *tyr.S* in each of the embryos generated by the experiment shown in Figure 4a, using the ICE analysis tool (Figure 4b, Figure S4). We found that the median *tyr.L*‐disruption ratios of the Condition IX, X, and XI embryos were 75.5%, 82.8%, and 70.5%, and their mean *tyr.L*‐disruption ratios were 75.0%, 80.0%, and 68.8%, respectively. The *tyr.L*‐disruption ratio was significantly higher in the Condition IX or X embryos compared to the Condition XI embryos. There was no significant difference in the *tyr.L*‐disruption ratios between the Condition IX and X embryos. In contrast to *tyr.L* whose disruption ratio varied at the different temperature conditions, *tyr.S* was efficiently disrupted at all the temperatures; the median disruption ratios of the Condition IX, X, and XI embryos were 98.9%, 90.9%, and 98.9%, respectively. Their mean disruption ratios were 88.0%, 87.9%, and 82.9%, respectively. There was no significant difference in the disruption ratios among these sample groups. These results suggest that the Condition IX allows one to disrupt target genes with high efficiency, without depending on the gene locus and/or a design of sgRNA. The lower disruption efficiency of the *tyr.L* locus may be related to the more condensed chromatin state of this locus compared to that of the *tyr.S* locus as indicated by ATAC‐seq analysis (http://www.xenbase.org/common/displayJBrowse.do?data=data/xl9_2). Although the 12°C incubation did not significantly increase the disruption efficiency, it may improve knock‐in efficiency in combination with our other optimized conditions (Kato et al., 2021).

The data presented in this study highlight the importance of balancing the experimental parameters to achieve the highest disruption efficiency. This includes accounting for the temperature dependency of the Cas9 nuclease activity, the time window for efficient gene disruption, and the temperature range that can be tolerated for the normal development of the species being used. These findings provide valuable insights into applying the CRISPR/Cas9‐mediated gene disruption technique to new experimental organisms.

## Supporting information


**Figure S1**. Pigmentation ratios of uninjected embryos cultured at different temperatures. A bar graph showing the pigmentation ratios of the uninjected embryos cultured at a constant temperature of 16, 22, or 25°C after fertilization, or initially at 12°C for 6 hours and subsequently at 22°C. The data are shown as in Figure 2. There were no significant differences in the pigmentation ratios among the sample groups
**Figure S2**. Reproducibility of the relationship in pigmentation ratios, culture conditions, and the rates of normally developed embryos. (A) In the bar graph, each dot indicates the mean pigmentation ratio for each group of embryos generated under Condition IX, X, or XI shown in Figure 4a. The sample groups were generated by three rounds of experiments, including the one shown in Figure 4a, and an additional two independent experiments using embryos obtained from different female and male pairs (Exp. 1, Exp. 2, and Exp. 3 shown in the table in [B], respectively). Each bar length represents the mean of the three means obtained from these three rounds of experiments. The error bars indicate SEM. Statistical analysis was performed using the Tukey–Kramer test (Tukey HSD) in R. (B) A table showing the number of embryos with normal external morphology and the total number of analyzed embryos for each sample group shown in the bar graph in A, along with the percentage of the embryos with normal external morphology. The bar graph represents the means of the percentages of normally developed embryos obtained from the three rounds of experiments for the sample groups shown in the table. The error bars indicate SEM. No significant differences were detected among the sample groups (shown as n.s.) by the TukeyKramer test.
**Figure S3**. Effects of varying the amount of injected Cas9 protein per embryo. A box‐and‐whisker plot showing the pigmentation ratios of the uninjected embryos (Uninj.) and the embryos generated under the experimental conditions shown in the Table (XII, XIII, and XIV) as dots. The data are shown as in Figures 2, 3, 4. There were no significant differences in the rates of normally developed embryos among the four sample groups.
**Figure S4**. Output data of the ICE analyses performed to analyze tyr.L and tyr.S disruption ratios in representative embryos generated under Conditions IX, X, and XI. The genomic sequences around the target regions of tyr.L‐sgRNA and tyr.S‐sgRNA are shown on the top, from left to right. The target sequences are underlined, and the PAM sequences are boxed in red. The tables in the left and right rows are the outputs of the ICE analyses that were performed for tyr.L and tyr.S loci, respectively, using representative embryos generated under the Conditions IX, X, or XI. A sum of the percentages of the indel patterns shown in each table indicates a total disruption ratio of the target gene in the analyzed representative embryo. The total disruption ratios of those embryos are indicated by black‐lined dots in Figure 4b
