# Supplementary material for: Optimization of CRISPR/Cas9‐mediated gene disruption in Xenopus laevis using a phenotypic image analysis technique
Source: Dev Growth Differ. 2022 Apr 12;64(4):219–25. doi: 10.1111/dgd.12778 (PMC11520957; doi:10.1111/dgd.12778)
Supplement: Supplementary file 1 — Figure S1. Pigmentation ratios of uninjected embryos cultured at different temperatures. A bar graph showing the pigmentation ratios of the uninjected embryos cultured at a constant temperature of 16, 22, or 25°C after fertilization, or initially at 12°C for 6 hours and subsequently at 22°C. The data are shown as in Figure 2. There were no significant differences in the pigmentation ratios among the sample groups Figure S2. Reproducibility of the relationship in pigmentation ratios, culture conditions, and the rates of normally developed embryos. (A) In the bar graph, each dot indicates the mean pigmentation ratio for each group of embryos generated under Condition IX, X, or XI shown in Figure 4a. The sample groups were generated by three rounds of experiments, including the one shown in Figure 4a, and an additional two independent experiments using embryos obtained from different female and male pairs (Exp. 1, Exp. 2, and Exp. 3 shown in the table in [B], respectively). Each bar length represents the mean of the three means obtained from these three rounds of experiments. The error bars indicate SEM. Statistical analysis was performed using the Tukey–Kramer test (Tukey HSD) in R. (B) A table showing the number of embryos with normal external morphology and the total number of analyzed embryos for each sample group shown in the bar graph in A, along with the percentage of the embryos with normal external morphology. The bar graph represents the means of the percentages of normally developed embryos obtained from the three rounds of experiments for the sample groups shown in the table. The error bars indicate SEM. No significant differences were detected among the sample groups (shown as n.s.) by the TukeyKramer test. Figure S3. Effects of varying the amount of injected Cas9 protein per embryo. A box‐and‐whisker plot showing the pigmentation ratios of the uninjected embryos (Uninj.) and the embryos generated under the experimental conditions shown in the Table (XII, [file DGD-64-219-s001.pdf]

Supplementary Materials

Optimization of CRISPR/Cas9-mediated gene disruption in *Xenopus laevis* using a phenotypic image analysis technique

Mikio Tanouchi[a], Takeshi Igawa[a], Nanoka Suzuki[a], Makoto Suzuki[a], Nusrat Hossain[a], Haruki Ochi[b] and Hajime Ogino[a*]

[a]Amphibian Research Center / Graduate School of Integrated Sciences for Life, Hiroshima University, 1-3-1 Kagami-yama, Higashi-Hiroshima, Hiroshima 739-8526, Japan.
[b]Institute for Promotion of Medical Science Research, Faculty of Medicine, Yamagata University, 2-2-2 Iida-Nishi, Yamagata 990-9585, Japan.

[*]**Author for correspondence**
E-mail: oginohaj@hiroshima-u.ac.jp
Phone: +81-82-424-7482
Fax: +81-82-424-0739

Supplementary Figures S1 to S4

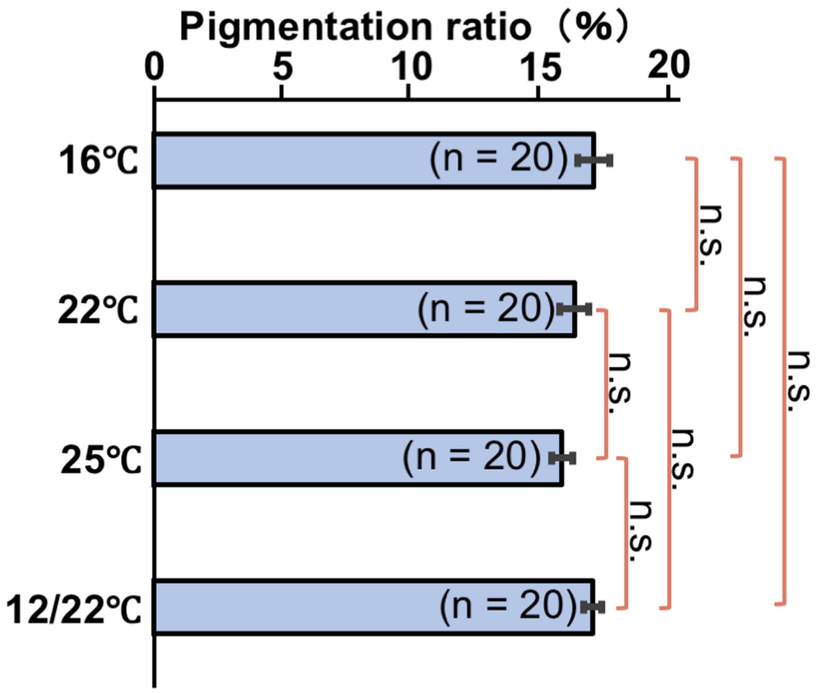

Fig. S1. Pigmentation ratios of uninjected embryos cultured at different temperatures. A bar graph showing the pigmentation ratios of the uninjected embryos cultured at a constant temperature of 16, 22, or 25°C after fertilization, or initially at 12°C for 6 hours and subsequently at 22°C. The data are shown as in Figure 2. There were no significant differences in the pigmentation ratios among the sample groups.

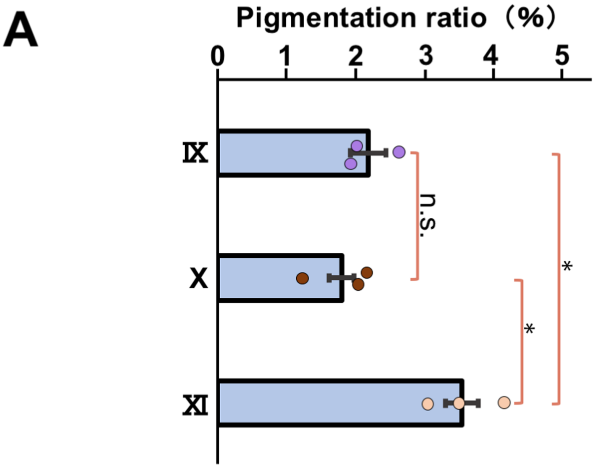

B

	Normally developed / total embryos (%)			
	Uninj.	IX	X	XI
Exp. 1	20/30 (66.7)	28/47 (59.6)	11/27 (40.7)	41/65 (63.1)
Exp. 2	28/30 (93.3)	42/51 (82.4)	24/47 (51.1)	57/69 (82.6)
Exp. 3	23/30 (76.7)	23/44 (52.3)	24/46 (52.2)	68/87 (78.2)

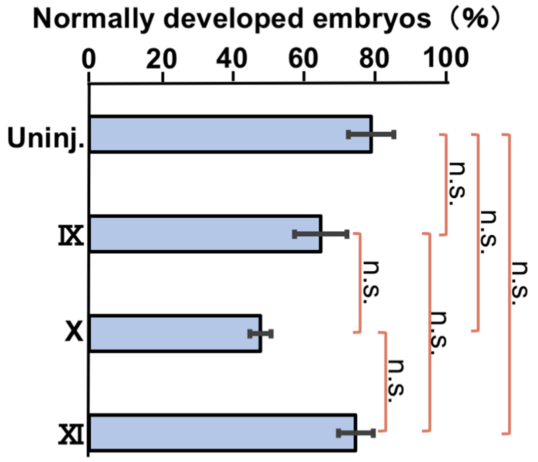

Fig. S2. Reproducibility of the relationship in pigmentation ratios, culture conditions, and the rates of normally developed embryos.

(A) In the bar graph, each dot indicates the mean pigmentation ratio for each group of embryos generated under Condition IX, X, or XI shown in Figure 4A. The sample groups were generated by three rounds of experiments, including the one shown in Figure 4A, and an additional two independent experiments using embryos obtained from different female and male pairs (Exp. 1, Exp. 2, and Exp. 3 shown in the table in (B), respectively). Each bar length represents the mean of the three means obtained from these three rounds of experiments. The error bars indicate SEM. Statistical analysis was performed using the Tukey-Kramer test (Tukey HSD) in R. (B) A table showing the number of embryos with normal external morphology and the total number of analyzed embryos for each sample group shown in the bar graph in A, along with the percentage of the embryos with normal external morphology. The bar graph represents the means of the percentages of normally developed embryos obtained from the three rounds of experiments for the sample groups shown in the table. The error bars indicate SEM. No significant differences were detected among the sample groups (shown as n.s.) by the Tukey-Kramer test.

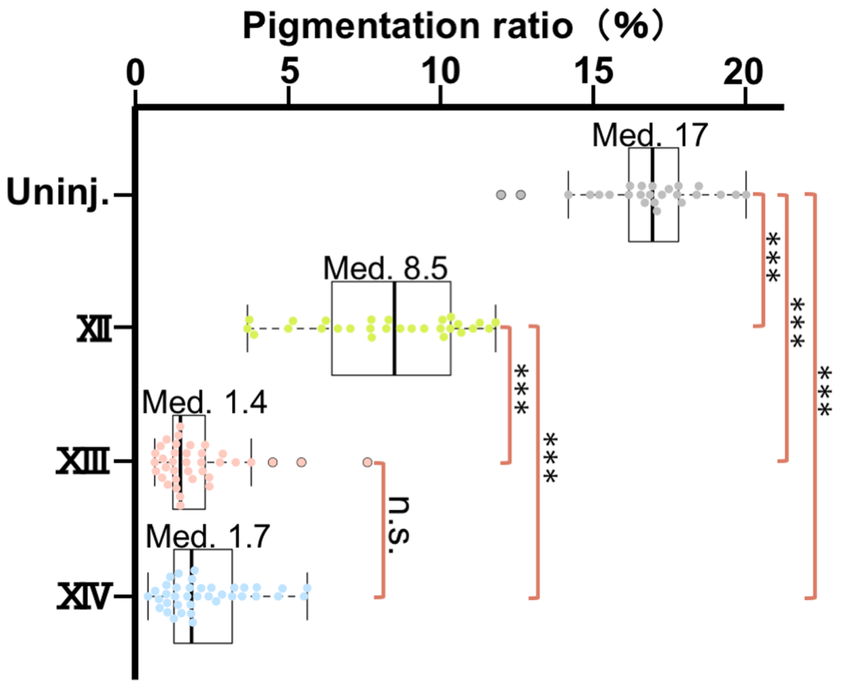

Condition	Injection timing (stage)	Cas9 protein (pg)	Culture temperature (℃)	Injection volume (nl)	Number of injection sites	Normally developed / total embryos (%)
Uninj.	-	-	22	-	-	25/30 (83.3)
XII	1-cell	1,000	22	20	2	29/43 (67.4)
XIII	1-cell	4,000	22	20	2	37/60 (61.7)
XIV	1-cell	8,000	22	20	2	38/53 (71.7)

Fig. S3. Effects of varying the amount of injected Cas9 protein per embryo.
A box-and-whisker plot showing the pigmentation ratios of the uninjected embryos (Uninj.) and the embryos generated under the experimental conditions shown in the table (XII, XIII, and XIV) as dots. The data are shown as in Figures 2–4. There were no significant differences in the rates of normally developed embryos among the four sample groups.

	tyr. L	*tyr. S*
	PAM (CCA) / Target sequences<br>CAGTGGGCCAGTC|CTCTCTATCATCGACCCCA	PAM (CCT) / Target sequences<br>ACGTCATCCTCAC|CAGCTCTGCTACGGGCCCT

IX — *tyr. L*

Indel numbers (bp)		Indel rates (%)
-3	CAGTGGGCCAGTC|---TCTATCATCGACCCCA	44.7
0	CAGTGGGCCAGTC|CTCTCTATCATCGACCCCA	24.5
-9	CAGTGGGCCAGTC|---------ATCGACCCCA	9.6
-5	CAGTGGGCCAGTC|-----TATCATCGACCCCA	6.4
-1	CAGTGGGCCAGTC|-TCTCTATCATCGACCCCA	5.3
-22	CAGTGG-------|---------------CCCA	4.3
+11	CAGTGGGCCAGTC|nnnnnnnnnnnCTCTCTATCATCGACCCCA	2.1
+1	CAGTGGGCCAGTC|nCTCTCTATCATCGACCCCA	1.1
+4	CAGTGGGCCAGTC|nnnnCTCTCTATCATCGACCCCA	1.1
+10	CAGTGGGCCAGTC|nnnnnnnnnnCTCTCTATCATCGACCCCA	1.1

IX — *tyr. S*

Indel numbers (bp)		Indel rates (%)
-2	ACGTCATCCTCAC|--GCTCTGCTACGGGCCCT	28.4
-1	ACGTCATCCTCAC|-AGCTCTGCTACGGGCCCT	21.6
-8	ACGTCATCCTCA-|-------GCTACGGGCCCT	12.5
-8	ACGTCATCCTC--|------TGCTACGGGCCCT	12.5
-15	ACGTCATCCTCA-|--------------GCCCT	11.4
-4	ACGTCATCCTC--|--GCTCTGCTACGGGCCCT	4.5
-6	ACGTCATCCTC--|----TCTGCTACGGGCCCT	2.3
0	ACGTCATCCTCAC|CAGCTCTGCTACGGGCCCT	1.1
-4	ACGTCATCCTCAC|----TCTGCTACGGGCCCT	1.1
-2	ACGTCATCCTCA-|-AGCTCTGCTACGGGCCCT	1.1
-5	ACGTCATCCTCA-|----TCTGCTACGGGCCCT	1.1
-7	ACGTCATCCTCA-|------TGCTACGGGCCCT	1.1
-10	ACGTCATCCTCA-|---------TACGGGCCCT	1.1

X — *tyr. L*

Indel numbers (bp)		Indel rates (%)
-3	CAGTGGGCCAGTC|---TCTATCATCGACCCCA	36.6
0	CAGTGGGCCAGTC|CTCTCTATCATCGACCCCA	17.2
-5	CAGTGGGCCAGTC|-----TATCATCGACCCCA	17.2
1	CAGTGGGCCAGTC|nCTCTCTATCATCGACCCCA	14.0
-8	CAGTGGGCCAGTC|--------CATCGACCCCA	9.7
-1	CAGTGGGCCAGTC|-TCTCTATCATCGACCCCA	1.1
-2	CAGTGGGCCAGTC|--CTCTATCATCGACCCCA	1.1
-9	CAGTGGGCCAGTC|---------ATCGACCCCA	1.1
-21	CAGTGGG------|---------------CCCA	1.1
+6	CAGTGGGCCAGTC|nnnnnnCTCTCTATCATCGACCCCA	1.1

X — *tyr. S*

Indel numbers (bp)		Indel rates (%)
-8	ACGTCATCCTC--|------TGCTACGGGCCCT	18.6
-25	ACG----------|---------------CCCT	12.8
-3	ACGTCATCCTCA-|--GCTCTGCTACGGGCCCT	8.1
-26	AC-----------|---------------CCCT	8.1
0	ACGTCATCCTCAC|CAGCTCTGCTACGGGCCCT	7.0
-4	ACGTCATCCTCAC|----TCTGCTACGGGCCCT	7.0
-8	ACGTCATCCTCA-|-------GCTACGGGCCCT	5.8
-27	A------------ ---------------CCCT	5.8
-3	ACGTCATCCTCAC|---CTCTGCTACGGGCCCT	4.7
-5	ACGTCATCCTCAC|-----CTGCTACGGGCCCT	3.5
-19	ACGTCATCC----|---------------CCCT	3.5
-11	ACGTCATCCTCAC|-----------CGGGCCCT	2.3
-14	ACGTCATCCTCAC|--------------GCCCT	2.3
-4	ACGTCATCCTC--|--GCTCTGCTACGGGCCCT	2.3
+1	ACGTCATCCTCAC|nCAGCTCTGCTACGGGCCCT	2.3
+2	ACGTCATCCTCAC|nnCAGCTCTGCTACGGGCCCT	2.3
-8	ACGTCATCCTCAC|--------CTACGGGCCCT	1.2
-14	ACGTCATCCTCA-|-------------GGCCCT	1.2
+13	ACGTCATCCTCAC|nnnnnnnnnnnnnCAGCTCTGCTACGGGCCCT	1.2

XI — *tyr. L*

Indel numbers (bp)		Indel rates (%)
-3	CAGTGGGCCAGTC|---TCTATCATCGACCCCA	42.1
0	CAGTGGGCCAGTC|CTCTCTATCATCGACCCCA	29.5
-5	CAGTGGGCCAGTC|-----TATCATCGACCCCA	9.5
-9	CAGTGGGCCAGTC|---------ATCGACCCCA	9.5
-1	CAGTGGGCCAGTC|-TCTCTATCATCGACCCCA	5.3
-4	CAGTGGGCCAGTC|----CTATCATCGACCCCA	1.1
-3	CAGTGGGCCA---|CTCTCTATCATCGACCCCA	1.1
-22	CAGTGG-------|---------------CCCA	1.1
+1	CAGTGGGCCAGTC|nCTCTCTATCATCGACCCCA	1.1

XI — *tyr. S*

Indel numbers (bp)		Indel rates (%)
-10	ACGTCATCCTCAC|----------ACGGGCCCT	23.6
+1	ACGTCATCCTCAC|nCAGCTCTGCTACGGGCCCT	23.6
-8	ACGTCATCCTC--|------TGCTACGGGCCCT	12.4
-11	ACGTCATCCTC--|---------TACGGGCCCT	7.9
-10	ACGTCATCCT---|-------GCTACGGGCCCT	6.7
-16	ACGTC--------|--------CTACGGGCCCT	5.6
-2	ACGTCATCCTCAC|--GCTCTGCTACGGGCCCT	4.5
-9	ACGTCATCCTCAC|---------TACGGGCCCT	4.5
-15	ACGTCATCCTCAC|---------------CCCT	3.4
-16	ACGTCATCCT---|-------------GGCCCT	2.2
0	ACGTCATCCTCAC|CAGCTCTGCTACGGGCCCT	1.1
-8	ACGTCATCCTCAC|--------CTACGGGCCCT	1.1
-2	ACGTCATCCTCA-|-AGCTCTGCTACGGGCCCT	1.1
-8	ACGTCATCCTCA-|-------GCTACGGGCCCT	1.1
-16	ACGTCATCCTCA-|---------------CCCT	1.1

Fig. S4. Output data of the ICE analyses performed to analyze *tyr.L* and *tyr.S* disruption ratios in representative embryos generated under Conditions IX, X, and XI.

The genomic sequences around the target regions of *tyr.L*-sgRNA and *tyr.S*-sgRNA are shown on the top, from left to right. The target sequences are underlined, and the PAM sequences are boxed in red. The tables in the left and right rows are the outputs of the ICE analyses that were performed for *tyr.L* and *tyr.S* loci, respectively, using representative embryos generated under the Conditions IX, X, or XI. A sum of the percentages of the indel patterns shown in each table indicates a total disruption ratio of the target gene in the analyzed representative embryo. The total disruption ratios of those embryos are indicated by black-lined dots in Figure 4B.
